# R‐COMP versus R‐CHOP as first‐line therapy for diffuse large B‐cell lymphoma in patients ≥60 years: Results of a randomized phase 2 study from the Spanish GELTAMO group

**DOI:** 10.1002/cam4.3730

**Published:** 2021-01-25

**Authors:** Juan‐Manuel Sancho, Rubén Fernández‐Alvarez, Francisco Gual‐Capllonch, Esther González‐García, Carlos Grande, Norma Gutiérrez, María‐Jesús Peñarrubia, Ana Batlle‐López, Eva González‐Barca, José‐María Guinea, Eva Gimeno, Francisco‐Javier Peñalver, Miguel Fuertes, Mariana Bastos, José‐Ángel Hernández‐Rivas, José‐María Moraleda, Olga García, Marc Sorigué, Alejandro Martin

**Affiliations:** ^1^ Hematology Department ICO‐IJC‐Hospital Germans Trias i Pujol Badalona Spain; ^2^ Hematology Department Hospital de Cabueñes Gijón Spain; ^3^ Cardiology Department of Hospital Germans Trias i Pujol Badalona Spain; ^4^ Hematology Department Hospital Doce de Octubre Madrid Spain; ^5^ Hematology Department Hospital Universitario de Salamanca IBSAL, CIBERONC Salamanca Spain; ^6^ Hematology Department Hospital Clínico de Valladolid Valladolid Spain; ^7^ Hematology Department Hospital Marqués de Valdecilla Santander Spain; ^8^ Hematology Department ICO‐Hospital Durán i Reynals (Hospitalet de Llobregat Barcelona Spain; ^9^ Hematology Department Hospital Universitario de Araba Vitoria Spain; ^10^ Hematology Department Hospital del Mar Barcelona Spain; ^11^ Hematology Department Hospital Universitario Fundación de Alcorcón Madrid Spain; ^12^ Hematology Department Hospital Clínico Lozano Blesa Zaragoza Spain; ^13^ Hematology Department Hospital Gregorio Marañón Madrid Spain; ^14^ Hematology Department Hospital Universitario Infanta Leonor Madrid Spain; ^15^ Hematology Department Hospital Virgen de la Arrixaca Murcia Spain

**Keywords:** cardiotoxicity, diffuse large B‐cell lymphoma, liposomal doxorubicin, N‐terminal pro‐B‐type natriuretic peptide, troponin

## Abstract

The use of non‐pegylated liposomal doxorubicin (Myocet^®^) in diffuse large B‐cell lymphoma (DLBCL) has been investigated in retrospective and single‐arm prospective studies. This was a prospective phase 2 trial of DLBCL patients ≥60 years old with left ventricular ejection fraction (LVEF) ≥55% randomized to standard R‐CHOP or investigational R‐COMP (with Myocet^®^ instead of conventional doxorubicin). The primary end point was to evaluate the differences in subclinical cardiotoxicity, defined as decrease in LVEF to <55% at the end of treatment. Secondary objectives were efficacy, safety, and variations of troponin and N‐terminal pro‐B‐type natriuretic peptide (NT‐proBNP) and LVEF along follow‐up.

Ninety patients were included, 45 in each group. No differences were observed in the percentage of patients with LVEF <55% at end of treatment (11% in R‐CHOP arm vs. 7% in R‐COMP arm, *p* = 0.697) or at 4 months (10% vs. 6%, respectively, *p* = 0.667) and 12 months (8% vs. 7%, respectively, *p* = 1). However, a higher percentage of R‐CHOP compared with R‐COMP patients showed increased troponin levels in cycle 6 (100% vs. 63%, *p* = 0.001) and at 1 month after treatment (88% vs. 56%, respectively, *p* = 0.015). Cardiovascular adverse events were seen in five R‐CHOP patients (nine episodes, four grade ≥3) and in four R‐COMP patients (five episodes, all grade 1–2). No significant differences in efficacy were observed.

In conclusion, R‐COMP is a feasible immunochemotherapy schedule for DLBCL patients ≥60 years, with similar efficacy to R‐CHOP. However, the use of non‐pegylated doxorubicin instead of conventional doxorubicin was not associated with less early cardiotoxicity, although some reduced cardiac safety signals were observed.

Trial registration: ClinicalTrials.gov Identifier: NCT02012088.

## Graphical abstract

The use of non‐pegylated liposomal doxorubicin (Myocet®) in diffuse large B‐cell lymphoma (DLBCL) has been investigated in retrospective and single‐arm prospective studies. This prospective randomized phase 2 trial of RCOMP versus RCHOP in DLBCL patients ≥60 years and normal cardiac function demonstrated that R‐COMP is a feasible immunochemotherapy schedule for DLBCL patients ≥60 years, with similar efficacy to R‐CHOP. However, the use of non‐pegylated doxorubicin instead of conventional doxorubicin was not associated with less early cardiotoxicity, although some reduced cardiac safety signals were observed.

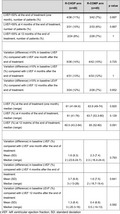

## 1 INTRODUCTION

The combination of the monoclonal anti‐CD20 antibody rituximab plus chemotherapy with cyclophosphamide, doxorubicin, vincristine, and prednisone (R‐CHOP) still constitutes the standard first‐line regimen for patients with diffuse large B‐cell lymphoma (DLBCL).^1^ However, its use is limited especially in elderly patients due, among other reasons, to cardiotoxicity derived from doxorubicin. Doxorubicin‐induced cardiotoxicity is caused by the binding of the drug and the ferric ion, producing the formation of free radicals that provides lipid peroxidation and progressive myocyte damage.^2^ The cumulative dose of doxorubicin appears to be the main factor involved in the development of cardiotoxicity, and although a threshold of 500 mg/m^2^ has been established as a risk dose, some studies have described early cardiotoxicity with doses of only 200 mg/m^2^.^3^, ^4^

In addition to clinical symptoms, determination of the left ventricular ejection fraction (LVEF) by echocardiography or cardiac scintigraphy (multi‐gated acquisition [MUGA]) scan is the most frequent parameter employed to evaluate cardiotoxicity, and usually a decrease in LVEF precedes the development of congestive heart failure.^3^ In recent years, the measurement of cardiac biomarkers, especially troponin and N‐terminal pro‐B‐type natriuretic peptide (NT‐proBNP), has been proposed as a method to detect early cardiotoxicity, and several studies have shown a relation between raised levels of cardiac biomarkers and left ventricular dysfunction, in particular with a decrease in LVEF, and the development of heart failure.^4^, ^5^, ^6^, ^7^, ^8^, ^9^

Several strategies have been proposed to decrease cardiotoxicity provoked by anthracyclines in elderly populations. These include the administration of reduced doses or slow infusions of doxorubicin, use of cardioprotective agents or substitution by other antineoplastic agents or by other less cardiotoxic anthacyclines, such as mitoxantrone, epirubicin, or liposomal formulations of doxorubicin.^10^, ^11^, ^12^, ^13^, ^14^ Myocet^®^ is a non‐pegylated liposomal doxorubicin that demonstrated to be less cardiotoxic—with similar antitumoral activity—than conventional doxorubicin in a phase 3 trial in patients with metastatic breast cancer.^2^ Due to its pharmacokinetic and pharmacodynamics profiles, it has also been associated with less myelosuppression and mucositis.^14^ However, its activity in lymphoma patients has mainly been investigated in retrospective and single‐arm prospective studies.^15^, ^16^, ^17^, ^18^, ^19^, ^20^

Taking into account this background, we designed a clinical trial for patients ≥60 years old diagnosed with DLBCL or grade 3b follicular lymphoma (FL) with normal cardiac function, with the main objective of evaluating the possible benefits in terms of cardiac toxicity, of the substitution of conventional doxorubicin by non‐pegylated liposomal doxorubicin (Myocet^®^, R‐COMP arm) as part of R‐CHOP therapy.

## 2 MATERIALS AND METHODS

Prospective randomized phase 2 trial (ClinicalTrials.gov Identifier: NCT02012088) of newly diagnosed patients ≥60 years old with non‐localized DLBCL or grade 3b FL with a baseline LVEF ≥55%. The trial was conducted according to Good Clinical Practice Guidelines and the 2008 revision of the Declaration of Helsinki. All patients provided written informed consent before participation in this study.

The requirements for the patients to be included were: age ≥60 years, newly diagnosed non‐localized DLBCL or grade 3b FL (those with localized lymphoma were included in the presence of bulky disease) with at least one measurable lesion, Eastern Cooperative Oncology Group (ECOG) performance status of 0–2, adequate hematological, renal and hepatic parameters (unless secondary to lymphoma involvement), and a baseline LVEF ≥55%. Patients with localized lymphoma, history of transformed lymphoma, central nervous system (CNS) involvement, or positivity for hepatitis B or C viruses or human immunodeficiency virus were excluded, as were those with clinically significant cardiovascular disease, such as non‐controlled arterial hypertension, non‐controlled ventricular or supraventricular arrhythmias, symptomatic ischemic heart disease (class II or higher according to the Canadian Cardiovascular Society criteria), past or present history of congestive heart failure, LVEF <55%, moderate to serious left ventricular hypertrophy, and significant valve abnormalities. In addition, patients with no adequate window to determine LVEF by echocardiography were also excluded.

Physical examination, standard blood tests, thoracic and abdominal computed tomography (CT) scan (and cervical if clinically indicated) plus positron emission tomography (PET) or combined PET/CT scan, and bone marrow biopsy were performed at baseline and at the end of treatment. For the cardiac evaluation, an electrocardiogram (ECG) and determination of the cardiac biomarkers troponin and NT‐proBNP in serum were performed at baseline, 48–72 h after the third and sixth cycles of chemotherapy and at the end of treatment (1 month after the last cycle of chemotherapy), and in the follow‐up visits performed 4 and 12 months later in each participant institution according to local procedures. The LVEF was measured by echocardiograpy at baseline, at the end of treatment (1 month after the last cycle of chemotherapy), and 4 and 12 months later. For LVEF determination, the biplane Simpson's method from the apical acoustic window was used,^21^ and the final result for the LVEF at each evaluation point was the mean of three measurements.

### 2.1 Treatment

Patients were randomized 1:1 to receive R‐CHOP (rituximab 375 mg/m^2^ [day 1], cyclophosphamide 750 mg/m^2^ [day 1], doxorubicin 50 mg/m^2^ [day 1], vincristine 1.4 mg/m^2^ [day 1, capped at a maximum of 2 mg], and prednisone 60 mg/m^2^ [days 1–5]) or R‐COMP (with the same drugs except for conventional doxorubicin being replaced by non‐pegylated liposomal doxorubicin, Myocet^®^, at doses of 50 mg/m^2^ [day 1]), administered in both arms every 21 days for a total of six cycles. If the delay in the administration of subsequent cycles was greater than 2 weeks due to toxicity, the patient was withdrawn from the study. Reductions of 25% and 50% in the doses of cyclophosphamide and doxorubicin (in R‐CHOP arm) or in the doses of cyclophosphamide and non‐pegylated liposomal doxorubicin (in R‐COMP arm) were mandatory for patients without hematological recovery (minimum neutrophil count of 1 × 10^9^/L and platelet count of 75 × 10^9^/L), after 1 or 2 weeks, respectively, of the 21‐day period of the previous cycle. In the case of grade 3–4 neuropathy, discontinuation of vincristine was mandatory, but the patients were allowed to continue to participate in the trial and receive the remaining drugs of the chemotherapy schedule. Primary prophylaxis of febrile neutropenia with granulocyte colony‐stimulating factor was allowed according to the clinical practice. CNS prophylaxis with intrathecal chemotherapy (according to clinical practice in the hospital) was recommended with each cycle of chemotherapy in the presence of increased serum lactate dehydrogenase plus involvement of more than one extranodal site, or in patients with a high International Prognostic Index (IPI) or in those with involvement of, at least, one of the following involved sites: paranasal sinus, Waldeyer's ring, epidural space, breast, kidney, or testes. Radiotherapy after chemotherapy on residual mass in patients with baseline bulky disease was also allowed according to the physician's decision.

### 2.2 Primary and secondary end points

The primary end point of the study was to evaluate the differences in subclinical cardiotoxicity, defined by a decrease in LVEF to <55% at the end of treatment (measured by echocardiography at 1 month after therapy), in patients receiving the standard R‐CHOP regimen compared with those treated with R‐COMP. Considering a non‐superiority hypothesis test for two independent samples with a statistical power of 80%, a significance level of 5% and assuming a proportion of subclinical cardiac toxicity in the reference and experimental groups of approximately 15%^22^, ^23^ and 5%,^22^, ^23^ respectively, with 5% dropouts, 45 patients in each treatment arm were necessary to be recruited.

Secondary end points were efficacy in terms of overall and complete response rates (ORR and CR) in all randomized patients, event‐free survival (EFS), progression‐free survival (PFS), overall survival (OS), and safety. Response to treatment was evaluated according to clinical, laboratory results and the evaluation of imaging techniques according to the criteria defined by Cheson et al.^24^ EFS was defined as the time from inclusion of the patients in the trial until treatment failure, including disease progression, treatment discontinuation, or death by any cause. PFS was defined as the time from inclusion into the trial until progression/relapse or death by any cause. OS was defined as time from study inclusion to death by any cause.

Safety, with special attention to cardiovascular toxicity, was evaluated according to clinical signs and laboratory parameters, and assessment of adverse events (AE) using version 4.0 of the NCI‐CTCAE scale for grading toxicity, as well as the variations in cardiac biomarkers troponin and NT‐proBNP in both arms throughout the study.

The primary end point analysis was carried out in patients who received six cycles of treatment and in whom post‐treatment evaluation of LVEF was performed. Efficacy analyses were carried out on an intention‐to‐treat (ITT) population, defined as all randomized patients, and in the evaluable population, defined as ITT population excluding patients who withdrew the trial without a response evaluation. The safety analysis was carried out in a safety population, which included all patients that received at least one cycle of chemotherapy.

### 2.3 Statistical analysis

Baseline demographic and clinical characteristics were described as frequency and percentage for categorical variables, and median and range for quantitative variables. Comparisons of categorical variables between treatment groups were studied using the Chi‐square test or Fisher's exact test, when necessary, while the median test was used to compare continuous variables.

For the primary end point (evaluation of differences in subclinical cardiotoxicity), the percentage of patients in whom the LVEF decreased to <55% at the end of treatment in each treatment group was compared using the Chi‐square test or Fisher's exact test, when necessary. The median LVEF at the end of treatment, as well as at 4 and 12 months later, and the median of the differences between baseline and end of treatment LVEF measures were compared by the nonparametric median test. Comparison of patients with variations in cardiac biomarkers throughout the study was made by Chi‐square test or Fisher's exact test, when necessary.

Regarding efficacy, OR and CR rates were compared using the Chi‐square test or Fisher's exact test, whereas EFS, PFS, and OS curves were plotted by the Kaplan–Meier method^25^ and compared by the log‐rank test.^26^

A descriptive analysis of the reported AE (frequency and percentage) was performed, and comparison between treatment groups was made using the Chi‐square test or Fisher's exact test.

No imputation method was used for missing data. Two‐sided *p* values <0.05 were considered as statistically significant. All analyses were performed with SPSS v24 (SPSS Inc.).

## 3 RESULTS

**FIGURE 1:**
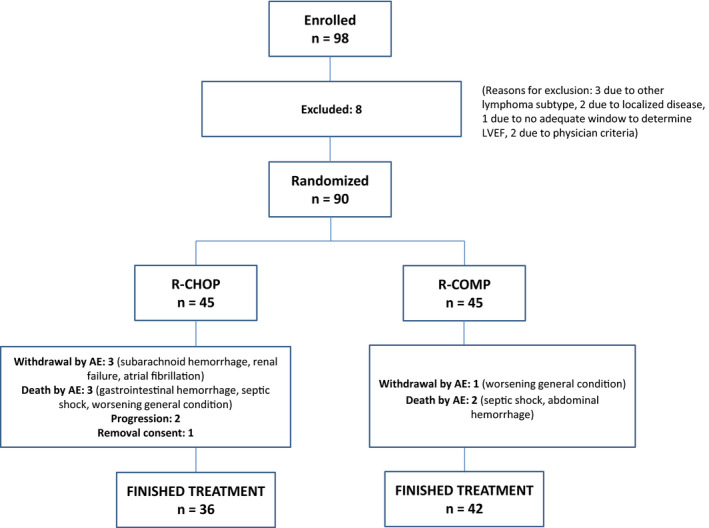
Flowchart of patients

From October 2013 to February 2016, a total of 90 patients with DLBCL from 15 hospitals belonging to the Spanish GELTAMO group were prospectively included. Of these, 45 were randomized to the R‐CHOP arm and 45 to the R‐COMP arm, without significant differences between the two arms regarding baseline characteristics (Table 1). The median age of the entire series was 74 years (range 60–86), with ECOG <2 in 83%; 79% of patients were in advanced stage and 42% had an intermediate to high IPI. The median LVEF at study entry was 64% (range 55–87.1), and almost half of patients had a previous history of hypertension. Figure 1 shows the flow chart of the patients along the study. Thirty‐eight out of 45 patients (84%) and 42/45 (93%) received six cycles of R‐CHOP and R‐COMP, respectively, (*p* = 0.130) and were included in the analysis of the primary end point.

**TABLE 1:** Baseline characteristics of the overall series and divided by treatment arm

	R‐CHOP arm (n = 45)	R‐COMP arm (n = 45)	*p* value	Overall series (n = 90)
Male, n (%)	17/45 (38%)	24/45 (53%)	0.138	41/90 (46%)
Age (years), median (range)	74 (60–84)	74 (60–86)	1	74 (60–86)
Baseline LVEF (%), median (range)	63 (55–81.4)	65 (55–87.1)	0.204	64 (55–87.1)
Hypertension, n (%)	26/45 (58%)	17/44 (39%)	0.071	43/89 (48%)
Diabetes, n (%)	7/45 (16%)	8/44 (18%)	0.741	15/89 (17%)
Dyslipemia, n (%)	21/45 (47%)	15/44 (34%)	0.227	36/89 (40%)
ECOG <2, n (%)	37/45 (82%)	38/45 (84%)	0.777	75/90 (83%)
B symptoms, n (%)	20/45 (44%)	19/44 (43%)	0.904	39/89 (44%)
Increased LDH level, n (%)	29/45 (64%)	23/45 (51%)	0.2	52/90 (58%)
Extranodal involvement, n (%)	26/45 (58%)	26/45 (58%)	1	52/90 (58%)
>1 extranodal site involved, n (%)	12/45 (27%)	13/45 (29%)	0.814	25/90 (28%)
BM involvement, n (%)	11/44 (25%)	12/45 (27%)	0.857	23/89 (26%)
Bulky disease, n (%)	11/44 (25%)	12/45 (27%)	0.857	23/89 (26%)
Ann‐Arbor stage, n (%)				
I–II	9/45 (20%)	10/45 (22%)	0.796	19/90 (21%)
III–IV	36/45 (80%)	35/45 (78%)		71/90 (79%)
IPI, (n %)				
0–2	25/44 (57%)	26/44 (59%)	0.829	51/88 (58%)
3–5	19/44 (43%)	18/44 (41%)		37/88 (42%)

Abbreviations: BM, bone marrow; ECOG, Eastern Cooperative Oncology Group; IPI, International Prognostic Index; LDH, lactate dehydrogenase; LVEF, left ventricular ejection fraction; WBC, white blood cells.

### 3.1 Subclinical cardiotoxicity: LVEF and cardiac biomarkers

Regarding the primary end point, no significant differences were observed between the R‐CHOP and R‐COMP arms in the number of patients with a LVEF <55% determined at the end (1 month) of treatment (11% [n =4/36] in the R‐CHOP arm vs. 7% [3/42] in the R‐COMP arm, *p* = 0.697), or at 4 months (10% [n =3/31] in the R‐CHOP arm vs. 6% [2/33] in the R‐COMP arm, *p* = 0.667) or at 12 months (8% [n =2/24] in the R‐CHOP arm vs. 7% [2/28] in the R‐COMP arm, *p* = 1) after therapy (Table 2). Furthermore, no differences were observed in an exploratory analysis comparing patients with a decrease in LVEF below 50% at the end of treatment or during follow‐up (data not shown), as well as in the number of patients with LVEF drop ≥10% at 1, 4, or 12 months after treatment compared to baseline LVEF. Similarly, there were also no significant differences between the two groups in the median LVEF determined at the end of treatment and at 4 and 12 months, or in the variations in LVEF determined at different times compared to the baseline LVEF (Table 2). Finally, no differences were detected in the primary end point (LVEF <55% at 1  month of treatment) considering all randomized patients (9% [n =4/45] in the R‐CHOP arm vs. 7% [3/45] in the R‐COMP arm, *p* = 1).

**TABLE 2:** Subclinical cardiotoxicity (LVEF) in both treatment arms throughout the treatment

	R‐CHOP arm (n = 45)	R‐COMP arm (n = 45)	*p* value
LVEF <55% at the end of treatment (1 month), number of patients (%)	4/36 (11%)	3/42 (7%)	0.697
LVEF <55% at 4 months of the end of treatment, number of patients (%)	3/31 (10%)	2/33 (6%)	0.667
LVEF <55% at 12 months of the end of treatment, number of patients (%)	2/24 (8%)	2/28 (7%)	1
Variation (difference) ≥10% in baseline LVEF (%) compared with LVEF 1 month after the end of treatment:	5/36 (14%)	4/42 (10%)	0.725
Variation (difference) ≥10% in baseline LVEF (%) compared with LVEF 4 months after the end of treatment:	4/31 (13%)	4/33 (12%)	1
Variation (difference) ≥10% in baseline LEVF (%) compared with LVEF 12 months after the end of treatment:	3/24 (12%)	2/28 (7%)	0.652
LVEF (%) at the end of treatment (1 month), median (range)	61 (41–84.6)	63.9 (49–74)	0.820
LVEF (%) at 4 months of the end of treatment, median (range)	61 (41–76)	63.7 (53.3–80)	0.129
LVEF (%) at 12 months of the end of treatment, median (range)	60.5 (43.2–84)	65 (52–80)	0.091
Variation (difference) in baseline LVEF (%) compared with LVEF 1 month after the end of treatment:			
Mean (SD)	1.6 (9.3)	2.3 (7.4)	0.793
Median (range)	2 (−23.6 to 24.7)	2.3 (−16.4 to 24.4)	
Variation (difference) in baseline LVEF (%) compared with LVEF 4 months after the end of treatment:			
Mean (SD)	3.7 (6.9)	1.6 (7.5)	0.841
Median (range)	3 (−13 to 28)	2 (−16.7 to 19.4)	
Variation (difference) in baseline LEVF (%) compared with LVEF 12 months after the end of treatment:			
Mean (SD)	1.3 (8.4)	0.4 (6.8)	0.592
Median (range)	3 (−20.3 to 16)	0.5 (−14 to 13)	

Abbreviations: LVEF, left ventricular ejection fraction; SD, standard deviation.

With respect to the cardiac biomarkers troponin and NT‐proBNP, a higher percentage of patients showed increased troponin levels (compared to baseline values) measured in cycle 6 (24/24 [100%] in the R‐CHOP arm vs. 17/27 [63%] in the R‐COMP arm, *p* = 0.001) and at 1 month after the end of treatment (21/24 [88%] in the R‐CHOP arm vs. 14/25 [56%] in the R‐COMP arm, *p* = 0.015), but not after cycles 3 or at 4 and 12 months after treatment (Table 3). Regarding NT‐proBNP, no differences were observed in the percentage of patients with increased levels along the treatment period (in cycles 3 and 6) and follow‐up (1, 4, and 12 months after therapy) compared to the baseline levels (Table 3).

**TABLE 3:** Cardiac biomarkers (troponin and NT‐proBNP) in both treatment arms throughout the treatment

	R‐CHOP arm (n = 45)	R‐COMP arm (n = 45)	*p* value
Troponin			
Increased troponin levels at cycle 3, number of patients (%)	13/23 (57%)	12/26 (46%)	0.469
Increased troponin levels at cycle 6, number of patients (%)	24/24 (100%)	17/27 (63%)	0.001
Increased troponin levels at end of treatment (1 month), number of patients (%)	21/24 (88%)	14/25 (56%)	0.015
Increased troponin levels at 4 months after treatment, number of patients (%)	16/21 (76%)	16/22 (73%)	0.795
Increased troponin levels at 12 months after treatment, number of patients (%)	10/15 (67%)	10/15 (67%)	1
NT‐proBNP			
Increased NT‐proBNP levels at cycle 3, number of patients (%)	25/29 (86%)	32/34 (94%)	0.401
Increased NT‐proBNP levels at cycle 6, number of patients (%)	27/29 (93%)	23/28 (82%)	0.253
Increased NT‐proBNP levels at end of treatment (1 month), number of patients (%)	14/29 (48%)	14/33 (42%)	0.644
Increased NT‐proBNP levels at 4 months after treatment, number of patients (%)	14/25 (56%)	12/27 (44%)	0.405
Increased NT‐proBNP levels at 12 months after treatment, number of patients (%)	12/18 (67%)	6/17 (35%)	0.063

Abbreviation: NT‐pro‐BNP, N‐terminal pro B‐type natriuretic peptide.

### 3.2 Efficacy evaluation

OR and CR were observed in 77 (85.5%) and 56 (62%) out of the 90 randomized patients, with no differences between treatment groups (OR and CR in the R‐CHOP arm in 36 [80%] and 28 [62%] patients, respectively; OR and CR in the R‐COMP arm in 41 [91%] and 28 [62%] patients, respectively). We performed an additional efficacy analysis including only the 80 patients evaluable for efficacy; 38 in the R‐CHOP arm (3 were excluded due to AE, 3 due to death from AE, and the remaining patient due to withdrawal of consent) and 42 in the R‐COMP arm (2 were excluded due to death from AE and the third patient due to AE). OR and CR were observed in 77 (96%) and 56 (70%) out of 80 evaluable patients, respectively, again without differences between the R‐CHOP and R‐COMP groups (ORR of 95% vs. 98%, *p* = 0.498, and CR rate of 74% vs. 67%, *p* = 0.494, respectively).

**FIGURE 2:**
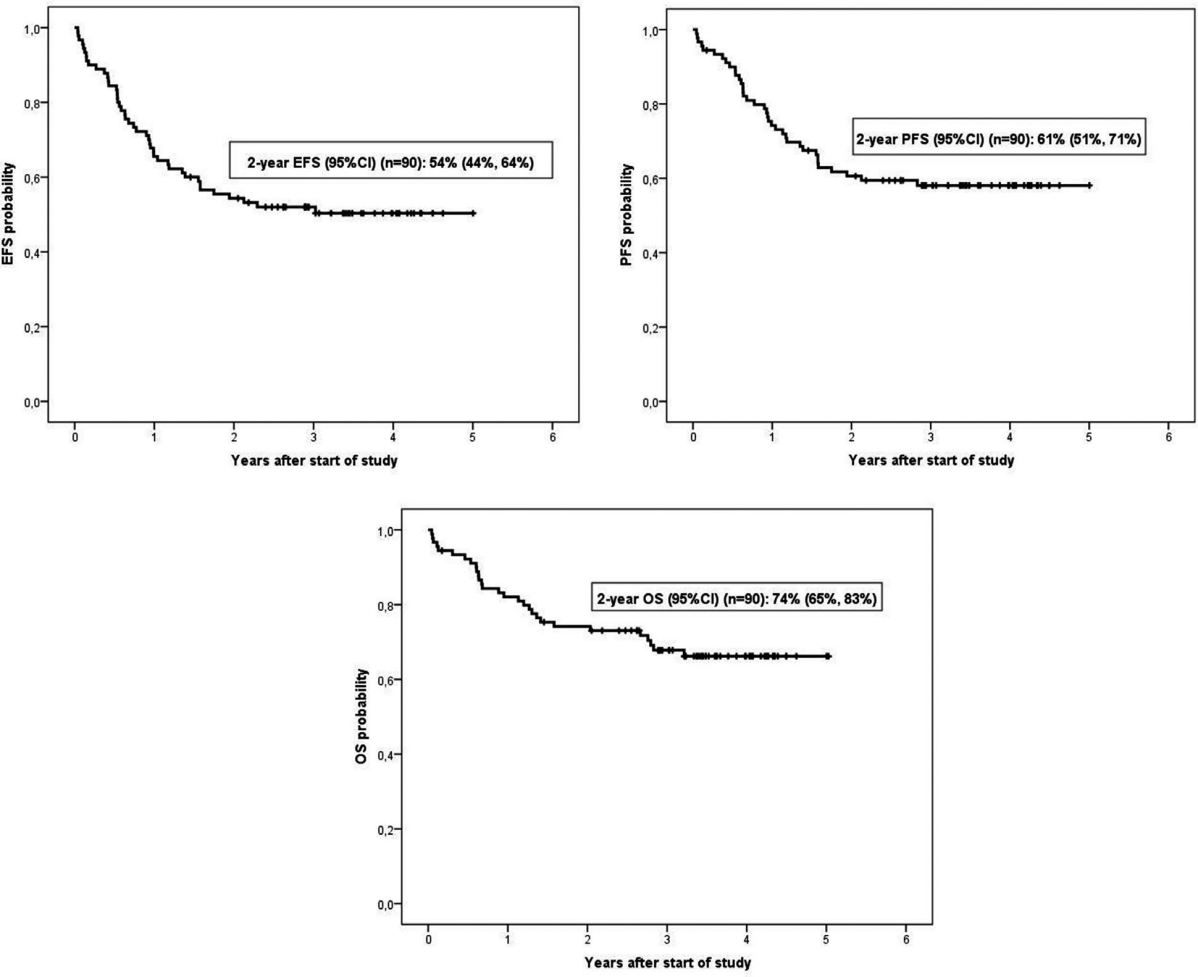
Event‐free survival (EFS), progression‐free survival (PFS), and overall survival (OS) probabilities for the overall series

**FIGURE 3:**
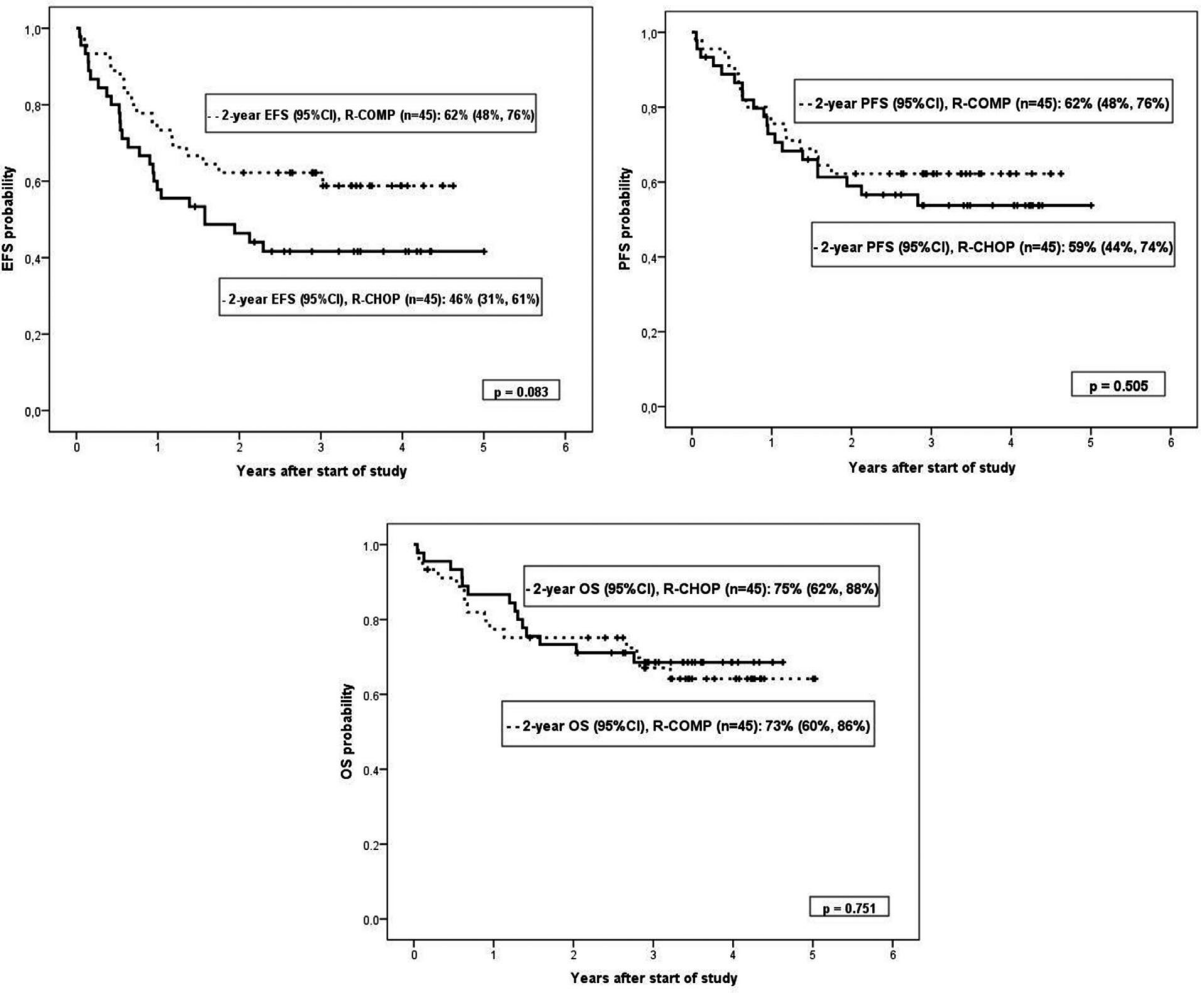
Event‐free survival (EFS), progression‐free survival (PFS), and overall survival (OS) probabilities by treatment group (RCHOP and R‐COMP groups represented in the continuous and dashed lines, respectively)

With a median follow‐up of 42 months (range 2.1–61.2) for patients alive at the time of analysis, 15 patients in the R‐CHOP group had died (6 by lymphoma) and 14 in the R‐COMP arm (10 by lymphoma).The 2‐year EFS and PFS probabilities for the entire series were 54% (95% confidence interval [CI] 44%–64%) and 61% (95% CI 51%–71%) (Figure 2), respectively, without differences between the two groups (2‐year EFS of 46% [95% CI 31%–61%] vs. 62% [95% CI 48%–76%] for R‐CHOP and R‐COMP patients, respectively, *p* = 0.083, and 2‐year PFS of 59% [95% CI 44%–74%] and 62% [95% CI 48%–76%] for RCHOP and R‐COMP patients, respectively, *p* = 0.505) (Figure 3).The 2‐year OS probability for the entire series was 74% (95% CI 65%–83%) (Figure 2), but again without significant differences between the two groups (75% [95% CI 62%–88%] for R‐CHOP patients vs. 73% [95% CI 60%–86%] for R‐COMP patients, *p* = 0.751) (Figure 3).

### 3.3 Safety

The main AEs reported by >5% of the patients are listed in Table 4. Overall, the most frequent non‐hematologic AEs were pain (53% of patients), fatigue (51%), infection (49%), peripheral neuropathy (31%), constipation (29%). and pyrexia (28%), with no differences between both arms. Non‐hematological grade 3–4 toxicity was also similar in both groups, being infection the most frequent (five patients [11%] in the R‐CHOP group and seven patients [16%] in the R‐COMP group). Regarding grade 3–4 hematological toxicity, neutropenia was observed more frequently in R‐CHOP patients (49% vs. 29%), but this was not translated into more incidence of febrile neutropenia, while thrombocytopenia and anemia were identical (Table 4).

**TABLE 4:** Number and percentage of patients with adverse events and toxicity (non‐hematologic and hematologic) that occurred in >5% patients in the R‐CHOP and R‐COMP arms

Non‐hematologic toxicity						
Adverse event/toxicity	Any grade				Grade 3–4	
	Overall series	R‐CHOP (n = 45)	R‐COMP (n = 45)	*p* value	R‐CHOP (n = 45)	R‐COMP (n = 45)
Pain	48 (53%)	24 (53%)	24 (53%)	1	1 (2%)	1 (2%)
Fatigue	46 (51%)	22 (49%)	24 (53%)	0.673	0	3 (7%)
Infection	44 (49%)	21 (47%)	23 (51%)	0.673	5 (11%)	7 (16%)
Peripheral neuropathy	28 (31%)	15 (33%)	13 (29%)	0.649	1 (2%)	0
Constipation	26 (29%)	13 (29%)	13 (29%)	1	1 (2%)	0
Pyrexia	25 (28%)	12 (27%)	13 (29%)	0.814	0	4 (9%)
Edema	23 (26%)	14 (31%)	9 (20%)	0.227	0	1 (2%)
Renal failure	21 (23%)	9 (20%)	12 (27%)	0.455	‐	‐
Diarrhea	20 (22%)	10 (22%)	10 (22%)	1	0	1 (2%)
Hepatic toxicity	18 (20%)	6 (13%)	12 (27%)	0.114	1 (2%)	3 (7%)
Nausea/vomiting	17 (19%)	8 (18%)	9 (20%)	0.788	‐	‐
Mucositis	16 (18%)	7 (16%)	9 (20%)	0.581	‐	‐
Hyperglucemia	14 (16%)	8 (18%)	6 (13%)	0.561	0	2 (4%)
Anorexia	13 (14%)	6 (13%)	7 (16%)	0.764	‐	‐
Dyspnea	13 (14%)	4 (9%)	9 (20%)	0.134	0	4 (9%)
Skin rash	13 (14%)	8 (18%)	5 (11%)	0.368	‐	‐
Hemorrhage	11 (12%)	5 (11%)	6 (13%)	0.748	1 (2%)	2 (4%)
Hypotension	7 (8%)	4 (9%)	3 (7%)	1	‐	‐
Dizziness	6 (7%)	2 (4%)	4 (9%)	0.677	‐	‐
Thrombosis	5 (6%)	2 (4%)	3 (7%)	1	1	0

Hematologic toxicity						
	Any grade				Grade 3–4	
	Overall series	R‐CHOP (n = 45)	R‐COMP (n = 45)	*p* value	R‐CHOP (n = 45)	R‐COMP (n = 45)
Neutropenia	46 (51%)	27 (60%)	19 (42%)	0.092	22 (49%)	13 (29%)
Febrile neutropenia	18 (20%)	7 (16%)	11 (24%)	0.292	7 (16%)	11 (24%)
Anemia	36 (40%)	18 (40%)	18 (40%)	1	3 (7%)	3 (7%)
Thrombocytopenia	16 (18%)	8 (18%)	8 (18%)	1	4 (9%)	4 (9%)

Cardiovascular toxicity is described in Table 5. Fourteen cardiovascular AEs were observed in nine patients, nine AEs in five patients who received R‐CHOP, and five AEs in four patients treated with R‐COMP. Four cardiovascular AEs were of grade ≥3 (two cases of atrial fibrillation, one heart failure, and one myocardial infarction), all of them in the R‐CHOP group.

**TABLE 5:** Cardiovascular adverse events in the R‐CHOP and R‐COMP arms

Adverse event	R‐CHOP (n = 45)	R‐COMP (n = 45)
Atrial fibrillation	3^a^	1
Tachycardia	3	1
Bradycardia/tachycardia	0	1
Heart failure	2^b^	1
Myocardial infarction	1^c^	0
Atrioventricular block	0	1
Overall cardiovascular events	9	5

^a^ Grade 3 in two cases.

^b^ Grade 3 in one case.

^c^ Causing death.

A total of 67 serious adverse events (SAEs) were reported in 39 patients (26 in 18 patients from the R‐CHOP group and 41 in 21 patients from the R‐COMP group), including 16 episodes of febrile neutropenia (6 in R‐CHOP and 10 in R‐COMP), 14 infections (7 in each group), 6 episodes of bleeding (2 in R‐CHOP and 4 in R‐COMP), and 4 episodes of pyrexia (all in the R‐COMP arm). Cardiovascular SAEs were reported in only five patients: supraventricular tachycardia (n = 2, R‐CHOP group), atrial fibrillation (n = 1, R‐COMP group), myocardial infarction (n = 1, R‐CHOP group), and heart failure (n = 1, R‐CHOP group).

## 4 DISCUSSION

This study demonstrates that non‐pegylated doxorubicin instead of conventional doxorubicin as part of the R‐CHOP regimen did not decrease the incidence of LVEF drop below 55% at the end of chemotherapy in patients ≥60 years old diagnosed with DLBCL with normal baseline cardiac function. Moreover, in this series, R‐COMP was a feasible immunochemotherapy schedule for patients ≥60 years of age with *de novo* DLBCL, with similar efficacy to R‐CHOP.

The main results of this study are concordant with a similar previous phase 3 trial by the Austrian AGMT group^23^ that compared R‐CHOP and R‐COMP in 79 adult patients with DLBCL and normal cardiac function. In the cited study, a low‐rate of cardiotoxicity was described in R‐CHOP and R‐COMP patients. However, while in the present study no significant differences were observed in the percentage of patients with LVEF <55% at the end of treatment (with only 11% and 7% of patients with a LVEF below <55% at the end of the study in R‐CHOP and R‐COMP patients, respectively), the Austrian group reported higher measurements of LVEF <50% throughout the study in R‐CHOP patients compared to the R‐COMP group (15.8% vs. 4.6%, respectively, *p* < 0.001), despite similar baseline LVEF values in both groups, a surprisingly finding since that younger patients were included in the Austrian (median age of 65 years and 38% of patients <60 years) compared to our study (median age of 74 years and all patients over 60 years old), and age is a well recognized risk factor for cardiotoxicity. However, as in the present study, no differences were observed in LVEF values at the end of treatment in the R‐CHOP compared to the R‐COMP group, suggesting that the substitution of conventional doxorubicin by non‐pegylated doxorubicin does not seem to protect against anthracycline‐derived subclinical cardiotoxicity, at least in terms of LVEF decrease in DLBCL patients. Similar results regarding LVEF variations were described previously in another phase 2 trial of 75 patients with DLBCL treated with 8 cycles of R‐COMP^20^; although LVEF measurements decreased at most time points, the differences were not significant, with a mean change from baseline to the end of treatment of −2.6%, very similar to that found in our study (Table 2). In addition, despite the lower number of measurements, the present study also suggests a lack of benefit in mid‐term subclinical cardiotoxicity, with similar LVEF measurements at 4 or at 12 months compared to baseline LVEF in the R‐CHOP and R‐COMP arms.

Cardiac biomarkers have been used as a complementary method to detect subclinical cardiac toxicity.^5^, ^6^, ^7^, ^8^, ^9^ Troponin and NT‐proBNP are related to early cardiac injury and heart failure, respectively. In the present study, only troponin levels more frequently increased in R‐CHOP compared to R‐COMP patients, although this increase was only detectable in cycle 6 and 1 month after the completion of treatment, but not at the other measurement times. This finding suggests the higher early cardiotoxicity in the R‐CHOP arm with the cumulative doses of conventional doxorubicin, but the absence of differences in troponin levels at other time points of follow‐up suggests later improvement. In fact, similar NT‐proBNP values were observed in both arms not only throughout treatment (after 3 and 6 cycles of treatment), but also during follow‐up (1, 4, and 12 months after therapy), conversely to the aforementioned study by Fridrik et al,^23^ in which higher NT‐proBNP values were detected in the R‐CHOP arm at cycle 6 and after treatment.

Overall, the frequency of cardiovascular and cardiac events was low in both arms despite the advanced age of the patients and long‐term follow‐up, with a total of 14 events (9 in R‐CHOP and 5 in R‐COMP), 4 of being grade ≥3, all of them in the R‐CHOP arm. These data are similar to those of other published studies.^16^, ^17^, ^20^, ^23^ In the phase 2 trial conducted by Luminari et al.,^20^ 15 out of 75 patients (21%) treated with R‐COMP (median age 72 years) presented cardiac AEs (grade 3–4 in 3 patients, corresponding to cardiac ischemia, atrial fibrillation, and congestive heart failure), whereas in the Austrian phase 3 trial,^23^ a total of 9 cardiac SAEs were observed, again without differences between R‐COMP (5 cardiac SAEs) and R‐CHOP (4 cardiac SAEs). Other studies with a lower number of patients treated with R‐COMP also described a low frequency of cardiac events.^16^, ^17^ These results are consistent with the previous large phase 3 randomized study by a French group^27^ that compared CHOP and R‐CHOP (eight cycles in each arm) in patients between 60 and 80 years old and described 8% of grade 3–4 cardiac events in the R‐CHOP arm, and with the randomized trial RICOVER‐60 by a German group,^28^ in which grade 3–4 cardiotoxicity was present in 3% of R‐CHOP‐14 patients (after six or eight cycles).

Non‐cardiovascular toxicity was also similar in both the R‐CHOP and R‐COMP groups, in contrast to some of the findings reported by Fridrik et al.^23^ In fact, whereas in the study by the Austrian group the number of SAEs was higher in R‐CHOP compared to R‐COMP patients (40 vs. 26, *p* = 0.029) due to increased infections, this was not the case in our study, with more SAEs being observed in the R‐COMP group because of febrile neutropenia, bleeding and pyrexia episodes, and despite a trend to higher incidence of neutropenia in R‐CHOP patients. In any case, this trend to higher neutropenia in the R‐CHOP group observed in the present study supports a possible lower hematological toxicity with liposomal doxorubicin compared to conventional doxorubicin due to its different pharmacokinetics.^14^ Moreover, no differences were observed in the frequency of mucositis, an AE that could decrease with liposomal doxorubicin according to other studies.^15^

As could be expected, efficacy was similar in both arms, in accordance with previously published data.^16^, ^19^, ^20^, ^23^ The CR rate was identical in both arms, but the ORR was higher in R‐COMP compared to R‐CHOP patients, although the differences were not significant. The EFS probability showed a trend to being higher in the R‐COMP arm, but PFS and OS were similar in both arms, and in line with other studies.^19^, ^20^, ^23^

There are some limitations in this study that should be mentioned. Primary end point was only assessed in 36/45 and 42/45 patients treated with R‐CHOP and R‐COMP, respectively, that exceeds the planned 5% rate of dropouts and could decrease the ability of the study to detect differences in subclinical cardiotoxicity, but in almost all cases discontinuations were due to non‐cardiac events. Moreover, LVEF evaluation was done in each participant institution, subject to interobserver variability, although all measurements were performed with the same method.^21^ However, in addition to the comparisons of the differences in LVEF between the two groups at the end of treatment, we also analyzed the variations in LVEF along the treatment, supporting the main findings of the study, which is also consistent with previous studies.^20^, ^23^ Global longitudinal strain (GLS) performed with echocardiography, another tool to detect subclinical cardiotoxicity, was not performed routinely. Similarly, troponin and NT‐proBNP biomarker measurements were not centralized, and thus, comparisons of cardiac biomarker values along the treatment and follow‐up were made with respect to baseline values in each patient. Nonetheless, the absence of significant variations of cardiac biomarkers supports the main finding of the study regarding the lack of significant LVEF changes in R‐CHOP‐ and R‐COMP‐treated patients. The incidence of cardiotoxicity was quite low in the present study, as it has been observed in other contemporary studies. However, a benefit of liposomal doxorubicin could be observed in selected patients with high risk of cardiotoxicity (excluded in the design of the study), such as those with previous anthracycline administration, borderline LVEF (50%–55%) or another type of heart disease with preserved LVEF.

In conclusion, this randomized trial confirms that the use of liposomal doxorubicin instead of conventional doxorubicin in the R‐CHOP regimen in older patients with DLBCL and a previously normal LVEF does not compromise the efficacy of the treatment, but neither does it appear to decrease early cardiac toxicity, at least in terms of the primary end point. Although we observed some signs of reduced cardiac toxicity in the R‐COMP group, short‐term cardiotoxicity was also low in the R‐CHOP group. Longer follow‐up and additional studies with a greater number of patients are needed to determine whether this drug could have benefits in late‐onset cardiac toxicity.

## CONFLICT OF INTEREST

JMS has received honoraria from Roche, Gilead, Janssen, Celgene, Servier, Novartis, Mundipharma, Sanofi, Kern‐Pharma, and Takeda, and was a Member of the Board of Directors or advisory committees for Roche, Janssen, Gilead, Celgene, Novartis, Bristol Myers, and Celltrion. NG has received honoraria from Roche and Janssen. EG has participated in advisory committees for Abbvie, Janssen, and Gilead. JAHR received honoraria and research funding from Roche, Janssen, Celgene, and Gilead. JMGDC received honoraria from Novartis and Alexion y Pfizer.

## Data Availability

The datasets used and/or analyzed during the current study are available from the corresponding author on reasonable request.

## References

[1] González‐Barca E , Coronado M , Martín A , et al; Spanish Lymphoma Group (GELTAMO) . Spanish Lymphoma Group (GELTAMO) guidelines for the diagnosis, staging, treatment, and follow‐up of diffuse large B‐cell lymphoma. Oncotarget. 2018;9:32383‐32399. 30190794 10.18632/oncotarget.25892 PMC6122355

[2] Batist G , Ramakrishnan G , Rao CS , et al. Reduced cardiotoxicity and preserved antitumor efficacy of liposome‐encapsulated doxorubicin and cyclophosphamide compared with conventional doxorubicin and cyclophosphamide in a randomized, multicenter trial of metastatic breast cancer. J Clin Oncol. 2001;19:1444‐1454. 11230490 10.1200/JCO.2001.19.5.1444

[3] Nousianen T , Jantunen E , Vanninen E , Hartikainen J . Early decline in left ventricular ejection fraction predicts doxorubicin cardiotoxicity in lymphoma patients. Br J Cancer. 2002;86:1697‐1700. 12087452 10.1038/sj.bjc.6600346 PMC2375393

[4] Limat S , Demesmay K , Voillat L , et al. Early cardiotoxicity of the CHOP regimen in aggressive non‐Hodgkin's lymphoma. Ann Oncol. 2003;14:277‐281. 12562656 10.1093/annonc/mdg070

[5] Cardinale D , Sandri MT , Colombo A , et al. Prognostic value of troponin I in cardiac risk stratification of cancer patients undergoing high‐dose chemotherapy. Circulation. 2004;109:2749‐2754. 15148277 10.1161/01.CIR.0000130926.51766.CC

[6] Cardinale D , Sandri MT . Role of biomarkers in chemotherapy‐induced cardiotoxicity. Prog Cardiovasc Dis. 2010;53:121‐129. 20728699 10.1016/j.pcad.2010.04.002

[7] Cardinale D , Salvatici M , Sandri MT . Role of biomarkers in cardioncology. Clin Chem Lab Med. 2011;49:1937‐1948. 21892906 10.1515/CCLM.2011.692

[8] Ky B , Carver JR . Biomarker approach to the detection and cardioprotective strategies during anthracycline chemotherapy. Heart Fail Clin. 2011;7:323‐331. 21749884 10.1016/j.hfc.2011.03.002

[9] Sandri MT , Salvatici M , Cardinale D , et al. N‐terminal pro‐B‐type natriuretic peptide after high‐dose chemotherapy: a marker predictive of cardiac dysfunction? Clin Chem. 2005;51:1405‐1410. 15932966 10.1373/clinchem.2005.050153

[10] Peyrade F , Jardin F , Thieblemont C , et al; Groupe dʼEtude des Lymphomes de lʼAdulte (GELA) investigators . Attenuated immunochemotherapy regimen (R‐miniCHOP) in elderly patients older than 80 years with diffuse large B‐cell lymphoma: a multicentre, single‐arm, phase 2 trial. Lancet Oncol. 2011;12:460‐468. 21482186 10.1016/S1470-2045(11)70069-9

[11] Yang F , Lei Q , Li L , et al. Delivery of epirubicin via slow infusion as a strategy to mitigate chemotherapy‐induced cardiotoxicity. PLoSONe. 2017;12:e0188025. 10.1371/journal.pone.0188025 PMC5683617 29131861

[12] Wiseman LR , Dexrazoxane SCM . A review of its use as a cardioprotective agent in patients receiving anthracycline‐based chemotherapy. Drugs. 1998;56:385‐403. 9777314 10.2165/00003495-199856030-00009

[13] Armitage JO . The role of mitoxantrone in non‐Hodgkin's lymphoma. Oncology (Willinston Park). 2002;16:490‐502. 12017536

[14] Allen TM , Martin FJ . Advantages of liposomal delivery systems for anthracyclines. Semin Oncol. 2004;31:161‐181. 15717735 10.1053/j.seminoncol.2004.08.001

[15] Levine AM , Tulpule A , Espina B , et al. Liposome‐encapsulated doxorubicin in combination with standard agents (cyclophosphamide, vincristine, prednisone) in patients with newly diagnosed AIDS‐related non‐Hodgkin's lymphoma: results of therapy and correlates of response. J Clin Oncol. 2004;22:2662‐2670. 15226333 10.1200/JCO.2004.10.093

[16] Rigacci L , Mappa S , Nassi L , et al. Liposome‐encapsulated doxorubicin in combination with cyclophosphamide, vincristine, prednisone and rituximab in patients with lymphoma and concurrent cardiac diseases or pre‐treated with anthracyclines. Hematol Oncol. 2007;25:198‐203. 17654614 10.1002/hon.827

[17] Visani G , Ferrara F , Alesiani F , et al. R‐COMP 21 for frail elderly patients with aggressive B‐cell non‐Hodgkin lymphoma: a pilot study. Leuk Lymphoma. 2008;49:1081‐1086. 18569635 10.1080/10428190802043853

[18] Moreno M , Sancho JM , Gardella S , et al. Non‐pegylated liposomal doxorubicin in combination with cyclophosphamide, vincristine, prednisone and rituximab for the treatment of non‐Hodgkin's lymphoma: study of 26 patients. Med Clin (Barc). 2010;134:72‐75. 19913261 10.1016/j.medcli.2009.05.042

[19] Mian M , Wasle I , Gamerith G , et al. R‐CHOP versus R‐COMP: are they really equally effective? Clin Oncol (R Coll Radiol). 2014;26:648‐652. 24929649 10.1016/j.clon.2014.05.012

[20] Luminari S , Montanini A , Caballero D , et al. Nonpegylated liposomal doxorubicin (Myocet^TM^) combination (R‐COMP) chemotherapy in elderly patients with diffuse large B‐cell lymphoma (DLBCL): results from the phase II EURO018 trial. Ann Oncol. 2010;21:1492‐1499. 20007997 10.1093/annonc/mdp544

[21] Lang RM , Badano LP , Mor‐Avi V , et al. Recommendations for cardiac chamber quantification by echocardiography in adults: an update from the American Society of Echocardiography and the European Association of Cardiovascular Imaging. Eur Heart J Cardiovasc Imaging. 2015;16:233‐270. 25712077 10.1093/ehjci/jev014

[22] Fridrik MA , Petzer AL , Keil F , et al. Non‐pegylated liposomal encapsulated doxorubicin reduces cardiotoxicity in 1st line treatment of diffuse large B‐cell lymphoma (DLBCL). Final results of a randomized trial. Blood. 2011;118(21):2676.

[23] Fridrik MA , Jaeger U , Petzer A , et al. Cardiotoxicity with rituximab, cyclophosphamide, non‐pegylated liposomal doxorubicin, vincristine and prednisolone compared to rituximab, cyclophosphamide, doxorubicin, vincristine, and prednisolone in frontline treatment of patients with diffuse large B‐cell lymphoma: a randomised phase‐III study from the Austrian Cancer Drug Therapy Working Group [ArbeitsgemeinschaftMedikamentöseTumortherapie AGMT](NHL‐14). Eur J Cancer. 2016;58:112‐121. 26990931 10.1016/j.ejca.2016.02.004

[24] Cheson BD , Pfistner B , Juweid ME , et al. Revised response criteriaformalignantlymphoma. J Clin Oncol. 2007;25:579‐586. 17242396 10.1200/JCO.2006.09.2403

[25] Kaplan GL , Meier P . Nonparametric estimation from incomplete observations. J Am Statist Assoc. 1958;53:457‐481.

[26] Peto R , Pike MC . Conservatism of the approximation (O‐E)2/E in the log‐rank test for survival data or tumour incidence data. Biometrics. 1973;29:579‐584. 4793138

[27] Coiffier B , Lepage E , Brière J , et al. CHOP chemotherapy plus rituximab compared with CHOP alone in elderly patients with diffuse large B‐cell lymphoma. N Eng J Med. 2002;346:235‐242. 10.1056/NEJMoa011795 11807147

[28] Pfreundschuh M , Schubert J , Ziepert M , et al. Six versus eight cycles of bi‐weekly CHOP‐14 with or without rituximab in elderly patients with aggressive CD20+ B‐cell lymphomas: a randomised controlled trial (RICOVER‐60). Lancet Oncol. 2008;9:105‐116. 18226581 10.1016/S1470-2045(08)70002-0

